# Cardiac contractility modulation in patients with heart failure: The added value of cardiac rehabilitation in identification, management, and follow-up

**DOI:** 10.1016/j.ijcrp.2024.200284

**Published:** 2024-05-16

**Authors:** Matteo Ruzzolini, Francesco Giallauria, Francesco Fattirolli, Elio Venturini, Francesco Maranta, Gian Francesco Mureddu, Pasqualina Calisi, Raffaele Griffo, Carlo Vigorito, Pompilio Faggiano, Marco Ambrosetti, Daniele Masarone

**Affiliations:** a Department of Cardiology, Isola Tiberina-Gemelli Isola Hospital, Rome, Italy, Italy; b Department of Translational Medical Sciences, "Federico II" University of Naples, Italy; c Department of Sperimental and Clinical Medicine, University of Florence, Italy; d Department of Cardiac Rehabilitation, Cecina, Italy; e Department of Cardiac Rehabilitation, IRCCS San Raffaele Hspital, Milan, Italy; f Department of Cardiac Rehabilitation, San Giovanni-Addolorata Hospital, Rome, Italy; g Cardiac Rehabilitation Unit, La Colletta Hospital, Arenzano, Italy; h Italian Alliance for Cardiovascular Rehabilitation and Prevention (ITACARE-P), Italy; i Cardiovascular Department, Fondazione Poliambulanza, 25100, Brescia, Italy; j Department of Cardiac Rehabilitation, ASST Crema, Rivolta d’Adda, Italy; k AORN Ospedale dei Colli, Napoli, IT, Italy

## 1 Introduction

Despite the developments of new drugs and devices, heart failure (HF) remains burdened by high morbidity and mortality, representing 1–2% of all hospital admissions in Europe and North America, with 1-year mortality of about 15–30 %. Consequently, the annual care costs amount to € 25,000 per patient, and a significant increase in cost is expected in the coming years, up to $53.1 billion in 2030 in the USA [1]. In addition, the increase in the average age of the population also brings with it an increase in comorbidities, including obesity, diabetes, and metabolic syndrome, which, together with the advances in HF treatment, have led to an increase in life expectancy as well as a better diagnosis capacity which provide to make an earlier diagnosis [2,3].

Theoretical best HF therapy is often not feasible due to side effects (e.g., symptomatic hypotension, hyperkalemia), which cannot allow to reach the maximum expected dosage in all patients [4,5]; conversely, only 30 % of all HF patients meet the criteria for receiving device therapy, such as cardiac resynchronization therapy (CRT) [6].

The new guidelines of the European Society of Cardiology (ESC) recommend early initiation of multiple guideline-directed medical therapies (GDMTs) to reduce mortality and worsening HF episodes in patients with HF with reduced ejection fraction (HFrEF) and mildy-reduced ejection fraction (HFmrEF) to improve adherence [7]. However, although some studies [8] have demonstrated the effectiveness of an early and “aggressive” approach, real-life data still show an unsatisfactory rate of prescription and titration of these drugs as well as inadequate adherence over time [9].

However, the peculiar characteristics of these patients often require a territorial organization that can guarantee regular and close follow-up, with a multidisciplinary approach as possible, for the evaluation and up-titration of the pharmacological therapy as well as the monitoring of possible side effects: the difficult full application of this model explains a non-optimal treatment for all patients treated for HF and the consequent still high number of hospitalizations and deaths caused by both cardiovascular and non-cardiovascular causes.

A recent Italian survey [10] involving 105 HF clinics showed that despite 94 % of patients receiving a regular follow-up every 3–6 months, available therapies were considered insufficient in 30 % of cases: physicians reported a lack of treatment options for 23 % of symptomatic patients with HF.

Cardiac Contractility Modulation (CCM) is a new device that gives further therapeutic opportunities for HFrEF and HFmrEF patients in this clinical scenario.

The 2021 ESC guidelines on HF set these goals for the management of patients with HF: improve symptoms and quality of life (QoL), achieve complete congestion relief, prevent early readmission, and improve survival; for this reasons, ESC guidelines consider CCM a device under evaluation to be considered in patients with NYHA class III-IV, LVEF ≥25 % to ≤45 % and QRS duration <130 ms, looking forward to further randomized clinical trials [11].

The 2022 American College of Cardiology/American Heart Association/Heart Failure Society of American Guideline for the Management of Heart Failure describes the CCM as a Food and Drugs Administration-approved device for patients with LVEF ≥25 % to ≤45 % who are not candidates for CRT, noting that effects on exercise capacity and QoL have been demonstrated but not on mortality or hospitalizations [12].

Therefore, aiming at identifying the ideal “responders” to CCM, some key elements have been proposed: a) NYHA class III despite optimal medical therapy; b) LVEF < or = to 45 %, LV end-diastolic diameter <70 mm, absence of systolic dysfunction of the right ventricle; c) arrhythmic burden with <8900 ventricular ectopic beats/24h; d) clinical stability (no HF re-acutization or hospitalizations in the previous month or absence of coronary events in the previous 3 months; e) absence of comorbidities affecting the prognosis quoad vitam at 6/12 months [13].

Cardiac rehabilitation (CR) settings can help to fill the gap in optimizing the diagnostic and therapeutic pathways of HF patients and “intercept all those who have an indication to upgrade their conditions, including electrical devices eligibility.

## 2 CCM: mechanisms of action

CCM is a device-based therapy for HF that involves applying electric signals to the right ventricular septal wall during the absolute myocardial refractory period. Accordingly, CCM signals do not elicit a new contraction; rather, they influence the biology of the failing myocardium and lead to several intracellular changes [14].

The CCM pulse is a train of 1–3 pulses with an amplitude of about 4.5–7.5 V and a phase length of about 5 ms, each pulse consisting of 2 phases of opposite polarity and programmable size.

The first devices used for CCM therapy required the detection of sinus rhythm, but new algorithms have been developed that permit the inclusion of patients with atrial fibrillation [15].

The stimulation cycles usually last 1 h and are used 7 times per day, each with breaks of 2–3 h. The device's battery has an average duration of 15 years, and the new device generation's battery has been extended to 20 years and is recharged by the patient once a week [16].

These electrical impulses improve myocardial function in different ways [17], also through reversion of cardiac maladaptive fetal gene program [18]: it improves calcium handling in cardiomyocytes, inducing beneficial molecular remodeling of intracellular calcium regulatory proteins [19]; it improves myofilaments interaction increasing phosphorylation of troponin and myosin [20]; it increases the expression of metalloproteinases counteracting fibrosis replacement of left ventricle [21]; it reduces the hyperactivation of sympathetic nervous system by stimulating vagal afferent fibers located in the septal wall [22].

In particular, the mechanisms that seem to best explain the effects of CCM therapy are [23,24].

**Fig. 1:**
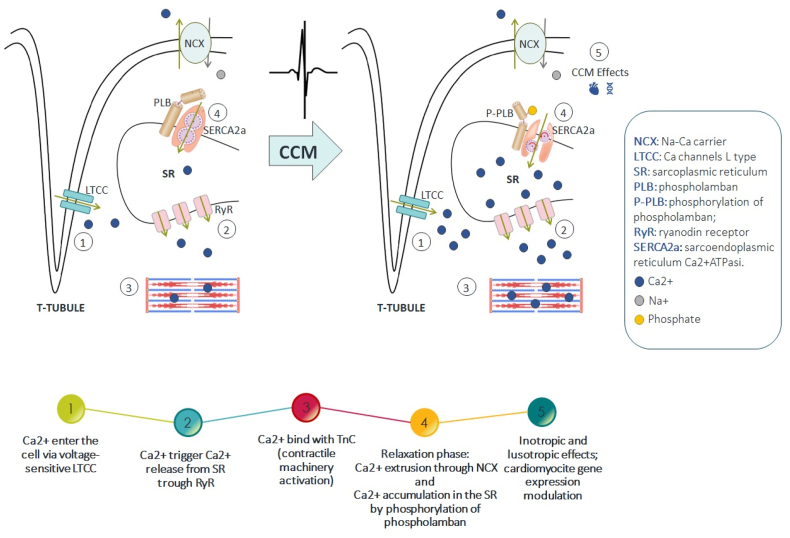
Molecular mechanisms of action of CCM.

- increase in the action potential's duration, capable of leading to an immediate increase in calcium, subsequently capable of enhancing the reuptake of the sarcoplasmic reticulum;
- enhancement of SERCA2a with chronic stimulation, partly already in the acute phase, mediated by phosphorylation of phospholamban, perhaps in the first few hours with an electron-mediated mechanism;
- changes in gene expression related to proteins involved in calcium contraction and reuptake mechanisms, most likely following chronic stimulation (Fig. 1).

If the acute local changes can, therefore, be partly explained by the modulation of ion channels, the mechanisms by which CCM therapy modifies gene expression by inducing reverse remodeling remain to be fully elucidated [13,25].

All these mechanisms result in improved calcium handling with enhancement of both systolic and diastolic filling function, reduced cardiac fibrosis, positive left and ventricular remodeling, and other effects resulting in improved functional capacity, QoL, and decreased hospitalizations.

## 3 Clinical evidence

Available clinical studies on CCM are affected by impressive heterogeneity; therefore, two synoptic tables synthesizing the different studies investigating CCM in various clinical scenarios and in different patients are reported (Table 1 and Table 2).

**Table 1:** Overview of relevant clinical studies investigating CCM.

Author	Year of publication	Device	N. of patients (M + F)	Randomized	Controlled (intervention in the control group)	Double-blinded	Mean Follow-up
Kuschyk J et al.	2021	Optimizer™ Smart System	503	No	No	No	24 months
Wiegn P et al. (FIX–HF–5C2)	2020	2-lead Optimizer™ Smart System	60 (53 + 7)	No	No (compared to FIX–HF–5C control group)	No	24 weeks
Kuschyk J et al.	2019	Optimizer™ III	17(14 + 3)	No	No	No	6 months
Abraham WT et al. (FIX–HF–5C)	2018	Optimizer™ IV (as descripted in the study rationale)^57^	160 (122 + 38)	Yes	Yes (OMT)	No	24 weeks
Röger S et al.	2016	Optimizer™ III and Optimizer™ IV	48 (45 + 3)	Yes	Yes (Two-lead vs One-lead delivery)	Yes	6 months
Kloppe A et al.	2016	OPTIMIZER™ system (not further specified)	19 (18 + 1)	Yes	Yes (5h/day vs 12h/day)	Yes	24 weeks
Liu M et al.	2016	Optimizer™ III	82 (70 + 12)	No	Yes (case control study)	No	75 vs 69 months (CCM vs control)
Kloppe A et al.	2016	Optimizer™ IV	68 (60 + 8)	No	No	No	4.5 years
Kuschyk J et al.	2015	Optimizer™ II and Optimizer™ III	81 (69 + 12)	No	No	No	34 months
Röger S et al.	2014	Optimizer™ IV	70 (60 + 10)	No	No	No	2.8 years
Kadish A et al. (FIX–HF–5)	2011	Optimizer™ III (as descripted in the study protocol)^28^	428 (309 + 119)	Yes	Yes (OMT)	No	6 months
Schau T et al.	2011	Optimizer™ II and Optimizer™ III	54 (49 + 5)	No	No	No	33 months
Yu CM et al.	2009	Optimizer™ III	30 (24 + 6)	No	No	No	3 months
Borggrefe MM et al. (FIX–HF–4)	2008	Optimizer™ II	181 (154 + 27)	Yes	Yes (device OFF)	Yes	6 months
Nägele H et al.	2008	Optimizer™ III	16 (12 + 4)	No	No	No	147 days
Neelagaru SB et al. (Pilot study for FIX–HF–5)	2006	OPTIMIZER™ system (not further specified)	49 (34 + 15)	Yes	Yes (device OFF)	Yes	6 months
Stix G et al. (FIX–HF–3)	2004	Optimizer™ II	25 (23 + 2)	No	No	No	8 weeks
Pappone C et al.	2002	Not specified	24 (15 + 9)	No	No (Dual-chamber pacing regarded as control)	No	No (acute study)
Pappone C et al.	2001	“SCEPTER”	15 (12 + 3)	No	No	No	No (acute study)

**Table 2:** Study design and major findings of clinical trials investigating CCM.

Study (Year of publication)	Design	Major Findings
Pappone C et al. (2001)	Acute feasibility study designed to assess cardiac haemodynamics of patients with heart failure in response to CCM signal delivery. Heart failure patients with EF below 35 % having either ischaemic or idiopathic dilated cardiomyopathy and where candidates for an EP study were included in the protocol.	Significant (p < 0.05) increases in LV + *d*p/*d*t_max_, LV systolic pressure and pulse pressure. No change in the rate of arrhythmias.
Pappone C et al. (2002)	Acute feasibility study with three different CCM protocols (respectively LV, RV stimulus and CCM + BVP) which included patients with either ischemic or idiopathic dilated cardiomyopathy and EF < 35 %, who were referred for an EP study or implantation of a pacing device	Both LV and RV CCM stimulation increased + *d*P/*d*t_max_ to a similar degree, with associated aortic pulse pressure increases (p < 0.01 vs controls). CCM signals delivered during biventricular pacing produced an additional increase in + dP/dtmax and in pulse pressure compared with BVP alone
Stix G et al. (FIX–HF–3, 2004)	First long-term feasibility study (8 weeks follow-up) which included patients with drug refractory NYHA class III heart failure and EF < 35 %.	Upon acute testing, the significant increase in + *d*P/*d*t_max_. EF and quality of life (MLHFQ) significantly improved (p = 0.0002 and p = 0.001 respectively). The 6MWT distance, performed in 7 patients at one of the participating centers, increased (p = 0.02).
Neelagaru SB et al. (Pilot study for FIX–HF–5, 2006)	Randomized, double-blind, pilot study conducted to determine the feasibility of safely and effectively delivering cardiac contractility modulation signals in patients with heart failure, EF < 35 % and NYHA III or IV despite medical therapy.	Compared with baseline, 6-min walk, peak VO2, and anaerobic threshold, increased more in the treatment group than in control (although the treatment group was considered “Sicker”). None of these differences were statistically significant (the authors considered the non-significance being due to small sample size). NYHA and Minnesota Living with Heart Failure Questionnaire changed similarly in the two groups.
Nägele H et al. (2008)	Feasibility study that explored CCM in CRT-NR patients, defined as patients remaining in NYHA classes III–IV despite optimized biventricular pacing and OMT.	Left ventricular + *d*P/*d*t_max_ measured in 14 patients out of 16 patients increased (p < 0.001) in the acute intraoperative testing. NYHA class and the EF improved at 3 months (both p < 0.01). No relevant electrical interference was observed between the CCM and CRT systems and no inadequate shocks were delivered in patients implanted with CRT-D.
Borggrefe MM et al. (FIX–HF–4, 2008)	Randomized, double blind, crossover study of cardiac contractility modulation (CCM) signals in heart failure patients older than 18 years, NYHA ≥2, ischaemic or idiopathic cardiomyopathy, EF ≤ 35 %, and peak VO_2_ between 10 and 20 mL O_2_/min/kg.	Statistically significant improvements in peak VO2 and MLHFQ (p ≤ 0.03 for each parameter) at the end of active treatment periods vs. end of sham treatment periods.
Yu CM et al. (2009)	Study that aimed to evaluate the impact of cardiac contractility modulation (CCM) on left ventricular (LV) size and myocardial function (evaluated by 3-dimensional echocardiography and TDI). TDI was also used to assess mechanical dyssynchrony.	LV reverse remodeling was evident, with a reduction in LV end-systolic volume and a gain in EF (both p < 0.001). Myocardial contraction was improved in all LV walls, including sites remote from CCM delivery (p = 0.05). TDI indexes showed improved systolic function and no changes in diastolic function and in dyssynchrony. Clinically, there was improvement of NYHA functional class and in 6MWT distance (p < 0.001 and p = 0.015 respectively). Premature ventricular contractions were not increased during CCM.
Schau T et al. (2011)	Retrospective study investigating the impact of CCM on cardiac and all-cause mortality on severe HF patients.	Data suggested no worsening of survival in the treatment of patients with end-stage heart failure by CCM.
Kadish A et al. (FIX–HF–5, 2011)	Randomized, unblinded, controlled trial comparing CCM to OMT.	CCM significantly improved peak VO_2_ and MLHFQ (p = 00.024 and p < 00.0001, respectively) over OMT. VAT did not improve at 6 months. Forty-eight percent of OMT and 52 % of CCM patients experienced a safety end point, which satisfied the noniferiority criterion (p = 0.03). Further adjunctive subgroup analysis by **Abraham et al.**^59^ based on this study's findings confirmed the hypothesis that CCM is more effective in patients with baseline EF ≥ 25 % and NYHA class III or lower)
Röger S et al. (2014)	Nonrandomized study conducted to assess the effect of CCM on intraventricular conduction (QRS duration)	No significant changes in mean QRS duration were measured comparing baseline to last follow up.
Kuschyk J et al. (2015)	Long-term single centre study analysing long-term efficacy and survival in patients with chronic heart failure treated with CCM.	CCM therapy improved quality of life (MLWHFQ), exercise capacity (peak VO_2_ and VAT), NYHA class, EF and NT-proBNP levels during long-term follow up. Mortality rates appeared to be lower than estimated from the MAGGIC score. All these changes were statistically significant.
Kloppe A et al. (2016)	Retrospective study evaluating survival in a cohort of CCM implanted patients with NYHA II or III symptoms and QRS duration ≤130 ms.	Mortality rates (Kaplan–Meier analysis) at 1-, 2- and 5-years were lower with CCM than predicted by SHFM for the cohort (p = 0.007).
Liu M et al. (2016)	Case-control study comparing patients affected by HF with an EF < 40 % who received CCM to patients with similar age, gender, EF, and aetiology of HF receiving standard treatment.	All-cause mortality was lower in the CCM group than the control group (p = 0.001). The improvement of all-cause mortality was more dramatic in patients with EF = 25–40 % (p < 0.001) than those with EF < 25 % (p = n.s.). Similar results were shown for the benefit of CCM in the secondary endpoints of cardiovascular death, and the composite outcome of death or heart failure hospitalization.
Kloppe A et al. (2016)	Randomized study comparing 5 versus 12 h per day of cardiac contractility modulation treatment for heart failure patients.	Clinical improvement was observed in the entire cohort in all efficacy measures (significant improvements in MLHFQ and NYHA, and non-significant improvements in peak VO_2_ 6MWD, and in EF). There were no significant differences, either clinically or statistically, between the groups receiving CCM for 5 h/day vs. 12 h/day.
Röger S et al. (2016)	Randomized comparison of signal delivery through one vs. two ventricular leads.	Following 6 months, similar and significant (p < 0.05) improvements from baseline in NYHA and MLWHFQ were observed in both groups. PeakVO_2_ showed improvement trends in both groups (p = ns). Serious adverse events occurrence was not different between groups. No statistically significant difference was found in any of the study endpoints.
Abraham WT et al. (FIX–HF–5C, 2018)	Randomized unblinded clinical trial that sought to confirm that CCM's efficacy is maximal in patients with EF between 25 % and 45 %. Patients with NYHA functional class III or IV symptoms, QRS duration <130 ms, and EF between 25 % and 45 % were randomized to continued medical therapy or CCM.	Peak VO_2_ improvements, Minnesota Living With Heart Failure questionnaire (p < 0.001), NYHA functional class (p < 0.001), and SMWT (p = 0.02) were all better in the treatment group after 24 weeks. The primary safety endpoint was satisfied (more than 70 % of patients had no device-related events). The composite of cardiovascular death and HF hospitalizations was reduced (p = 0.048).
Kuschyk J et al. (2019)	Non- randomized unblinded study evaluating CCM in CRT non-responders	Peak VO_2_ increased (p = 0.03) and MLWHFQ improved (p = 0.01). Mean NYHA class improved (p = 0.02), 6MWT increased (p < 0.01), while EF trended up (p = 0.08) at 6 months.
Wiegn P et al. (FIX–HF–5C2, 2020)	Non-randomized unblinded study evaluating safety, performance, and efficacy of CCM delivered by the 2-Lead Optimizer Smart System.	CCM delivery did not differ significantly between 2- and 3-lead systems (comparable number of CCM signals/day). The change of peak VO2 from baseline to 24 weeks was 1.72 (95 % Bayesian credible interval, 1.02–2.42) mL/kg per minute greater in the 2-lead device group versus controls. More subjects in the 2-lead group experienced ≥1 class New York Heart Association improvement (p < 0.001). There were decreased Optimizer-related adverse events with the 2-lead system compared with the 3-lead system (p = 0.03).
Contaldi C.et al. (2020)	Study on the effects of CCM on RV systolic function and RV–pulmonary artery (PA) coupling	At six months follow up, CCM therapy increased RV performance, improving RV systolic function, PASP, and coupling between RV and PA. A better forward ejection of blood could be useful for RV reverse remodeling.
Masarone D. et al. (2022)	Evaluation on the effects of CCM on myocardial mechano-energetic efficiency (MEE) and global longitudinal strain (GLS)	At six months of follow-up, CCM therapy increased left ventricular performance, improving left ventricular ejection fraction, E/e’ ratio, GLS, as well as MEE and MEEi in patients with HFrEF on optimal medical therapy. These echocardiographic improvements are associated with a clear clinical benefit documented by reduction of NT-pro BNP plasma levels NYHA class and MLHFQ score.
Fastner C. et al. (Maintained Observational Study 2022)	Evaluation of long-term effects of CCM in patients with baseline NYHA class II versus baseline NYHA class III or ambulatory IV from clinical registry MAINTAINED Observational Study	In clinical practice, CCM was infrequently performed in NYHA class II patients. No significant improvement in NYHA class/dyspnea was observed in these patients over 5 years. Because of the improvement in LVEF, sustainable positive effects on long-term cardiac reverse remodeling might be expected in young patients. Patients with advanced heart failure showed improvements in NYHA class, LVEF, and TAPSE also in clinical practice.

**BVP**: Biventricular Pacing; **EP**: Electrophysiologic; **LV**: Left Ventricle; **RV**: Right Ventricle; **CRT-NR**: Cardiac Resynchronization Therapy - Non-Responders; **OMT**: Optimal medical therapy; **MAGGIC**: Meta-Analysis Global Group in Chronic; **SHFM**: Seattle Heart Failure Model; **Peak VO**_**2**_: Peak Oxygen uptake; **+ dP/dt**_**max**_: maximal rate of rise of pressure; **EF**: left ventricular Ejection Fraction; **MLHFQ**: Minnesota Living with Heart Failure Questionnaire; **VAT**: Ventilatory Anaerobic Threshold; **6MWT**: 6 Minute Walk Test.

In Table 1 is reported an overview of relevant clinical studies investigating CCM. Notably, these studies have many different features such as criteria of inclusion and exclusion, duration of follow-up, type of device, type of recruitment, CCM stimulation protocol, blinding or unblinding, presence of control group, type of treatment in the control group, outcomes measured and sample size (Table 1). Table 2 showed study design and major findings of clinical trials investigating CCM (Table 2)

Briefly, The FIX–HF–5C trial, a randomized multicenter clinical trial conducted in 2018, evaluated the efficacy and safety of CCM in patients with chronic HF. Results demonstrated significant improvement in exercise capacity (pVO2 and 6MWT), QoL (MLWHF), improvement of at least one NYHA class, with better results achieved in the group with LVEF 35–45 %.

FIX–HF–5C2: A further randomized study conducted in 2020 examined the long-term effect of CCM in patients with chronic HF. Results demonstrated persistent improvements in exercise capacity, QoL, and cardiac function after 24 months of follow-up.

A systematic review and meta-analysis published in 2020 by Giallauria et coll. analyzed individual data from RCT studies on CCM showing a significant improvement in exercise capacity, LVEF, and QoL in CCM-treated patients compared to controls [26]. A meta-analysis of patients’ data from all known randomized trials in 2020 has shown that CCM provides statistically significant and clinically benefits in functional capacity and HF-related quality of life [27].

The CCM-REG [28], a real-world registry of 140 patients published by Anker et coll. in 2019, showed a significant reduction in hospitalizations for HF and other cardiovascular causes in 2 years of follow-up.

The CCM-REG is a prospective registry study including 503 patients from 51 European centers published by Kuschyk et coll. in 2021. Effects were evaluated in three terciles of LVEF (≤25 %, 26–34 % and ≥35 %) and in patients with atrial fibrillation (AF) and normal sinus rhythm (NSR). Cardiac contractility modulation therapy improved functional status, quality of life, LVEF and, compared to patients’ prior history, reduced heart failure hospitalization rates during 2-year follow-up [29].

Other studies confirmed these positive effects of CCM therapy, and some others also investigated beneficial effects in increasing LVEF without an increased myocardial oxygen consumption [30,31], reduction of NTproBNP levels, CRT non-responders patients [32,33], on right ventricular function [34,35], and in patients with heart failure with preserved ejection fraction [36,37].

Table 1, Table 2 offer an overview of relevant clinical studies and trials investigating CCM.

Finally a recent study evaluated the cost-effectiveness of CCM therapy plus optimal medical therapy (OMT) compared to OMT alone in patients with heart failure with reduced ejection fraction [38]. This analysis reported very positive results, particularly: the base case results showed that the CCM plus OMT option was highly cost-effective compared with OMT alone with an incremental cost–utility ratio of €7034/quality-adjusted life year (QALY).

The CEAC and CEAF illustrated that for all willingness to pay levels above €5600/QALY, tested up to €50 000/QALY, CCM plus OMT alternative had the highest probability of being cost-effective.

The analysis demonstrated that implementing CCM therapy plus OMT over a lifetime period would be cost-effective at a threshold of €30 000 in the Italian National Health System. In sensitivity analysis, the model results were robust to most assumptions and parameter uncertainty.

These results show that the use of CCM in heart failure patients and NYHA III class at baseline is likely to be cost saving at the current price, in terms of healthcare costs.

## 4 From implantation to CCM management: the added value of cardiac rehabilitation programs

CR programs are strongly recommended (Class IA) in patients with established HF regardless of LVEF and the presence of cardiac implantable electronic or ventricular assistant devices, primarily for the multidisciplinary approach. ESC Guidelines recommends beginning as soon as possible CR programs in those patients followed by a structured outpatient CR program, which is crucial to improving patients’ exercise capacity and symptoms, improving QoL and prognosis (i.e., worsening HF episodes) [39].

CR programs are also provided to specific populations such as the elderly, frail people, obese and cancer patients and, today, with specific settings like telerehabilitation [40] to reach not only the rural population but also people who can't reach hospital services for any other personal/physical problems.

A recent EAPC position paper [41] has updated the practical recommendations on the core components of cardiac rehabilitation intervention in different cardiovascular conditions, defined as specific areas of intervention in the context of multidisciplinary structured cardiac rehabilitation activities aimed at obtaining clinical stabilization, cardiovascular risk reduction, disability reduction, psychosocial and vocational support, and lifestyle behavioral change including patients’ adherence and self-management.

Concerning exercise training, emphasis was put on the systematic adoption of the FITT (frequency, intensity, time duration, and type of exercise) prescription model. Type should also include the mode of training (i.e., the endurance continuous or interval modality for aerobic training, or the involvement of muscular groups for resistance/strength training), as far as leisure activities to meet patients’ preferences.

Cardiac rehabilitation also represents a particular and precious moment for optimizing pharmacological therapy (titration, onset of new drugs after clinical stabilization, monitoring possible side effects) and providing indications for eligibility for electrical therapies, including CCM; consequently, to all parameters evaluated in this phase as well as to the other evaluation performed. To date the added value of exercise-based CR after CCM implantation has never been estimated.

## 5 Optimizing patient's selection for CCM therapy: role of the Italian alliance for cardiovascular rehabilitation and prevention (ITACARE-P)

In the 2021 ESC Guidelines on HF [42], the CCM is cited as a device that could be used in those symptomatic patients (NYHA class III/IV), reduced left ventricular systolic function, optimized medical therapy, and QRS interval <130 msec on the electrocardiogram to improve symptoms, exercise tolerance, QoL and reduction in hospitalization; also, CRT non-responder patients are considered eligible.

Several articles have proposed operative flow charts just to describe the most suitable path to select patients who are candidates for CCM therapy considering the right patient, at the right time, and in the right clinical conditions.

Particularly, it was recently proposed by Masarone et al. [43] the HOPE algorithm aimed to simplify the selection of the patient candidate for CCM implantation using clinical and echocardiographic parameters easily obtainable in the common clinical practice to be performed step by step: the importance of the index event in determining the symptoms, functional capacity and quality of life (H); the optimized medical therapy (O), the absence of comorbidities that can negatively affect the effectiveness of the CCM (P), the confirmation of an EF between 25 and 45 % on the echocardiogram (E).

Once all these conditions are satisfied, the patient is eligible for CCM therapy, and is likely to obtain the best benefits.

These selection criteria could also be easily evaluated when patients are referred to CR and during CR programs. This would allow early identification of patients who are potential candidates for CCM implantation in the broader context of optimizing and personalizing the whole therapy for HF and subsequently referring the patient to a reference Electrophysiology (EP) Center for the implantation with correct timing and reduction waiting times just to benefit CCM therapy effects as soon as possible.

In this scenario, ITACAREP could promote a network between CR and EP centers, integrating CR and EP cardiologists, enhancing selection data, and optimizing times for implantation.

The selection of the patient in the CR setting could represent an added value considering specific tools proper to the CR activity, for example, the functional evaluation by using a cardiopulmonary test or 6MWT (even re-executable several times) that, together with the possibility of optimizing therapy for HF over a longer period of time compared to acute hospitalization and the evaluations and activities of physiotherapist, nutritionist, and psychologist can lead to a better selection of the CCM eligible patient, defined as the one who greater can benefit from CCM therapy and correct interventional timing.

Furthermore, CR programs phases 3 and 4 (outpatient and home-based) could re-evaluate all those patients who "missed" an initial evaluation in the acute setting or those considered not eligible in that specific heart failure context and time.

CR setting may also implement the evaluations and measurement of the effects of CCM therapy: clinical and echocardiographic re-evaluation, reassessment of functional capacity (cardiopulmonary exercise stress testing or 6MWT), quality of life and psychophysical well-being questionnaires that better define and quantify the results obtained by CCM therapy. Since the multi-comprehensive approach granted by clinical cardiologist, electrophysiologist, exercise physiologist and nurses, CR setting might represent an ideal opportunity for the best management for CCM patients.

**Fig. 2:**
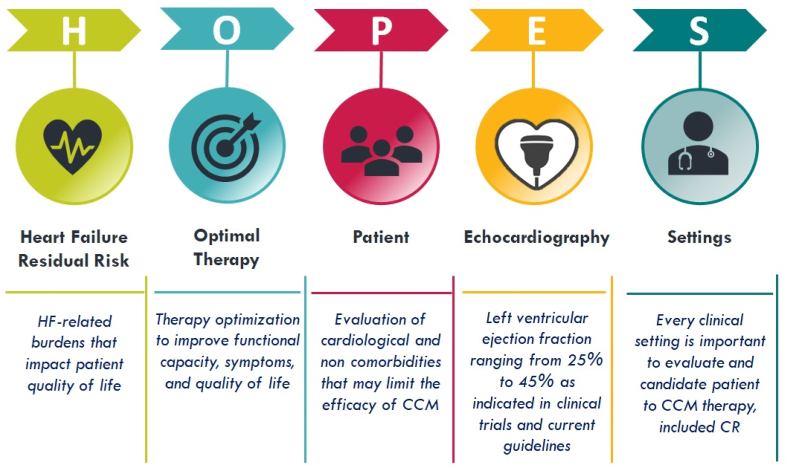
HOPES algorithm to simplify CCM patient selection.

For this reason, a new acronym could be suggested by extending that one already proposed by adding the "S" to the current HOPE algorithm forming the new word "HOPES", where the "S" stands for “setting,” indicating the possibility of carrying out all eligibility assessments of the patient both in acute inpatient or outpatient context (as already happens) and in the CR phases, in all its applications, thus becoming equally important as the other evaluations; from the “setting” as seen, strongly depends application time of CCM therapy (Fig. 2).

In addition, ITACAREP may favor a functional network between acute cardiologists, CR, and territorial care settings to implement HF therapy optimization, including CCM therapy, to improve clinical conditions, reduce rehospitalizations, and improve quality of life. In this scenario, CR can also offer an effective contribution to the collection of important data for the evaluation of the effects of these new application on the most important endpoints, thanks to the development of this "early evaluation" process.

Furthermore, in the light of the latest developments in research on particular pathologies that cause HF which have become the subject of CCM implantation with benefit (cardiomyopathy due to laminopathy, HF with preserved EF, right HF, cardiac amyloidosis), early identification of these patients as well as the possibility of closer follow-up as well as dedicated and personalized rehabilitation programs repeated over time could lead to further benefits in terms of functional capacity, exercise tolerance and quality of life.

## 6 Conclusions

Despite recent advances in pathophysiology understanding and pharmaceutical treatments, HF is still burdened by a high mortality and costs related.

CCM therapy might be crucial for improving QoL and exercise tolerance and reducing hospitalizations.

**Fig. 3:**
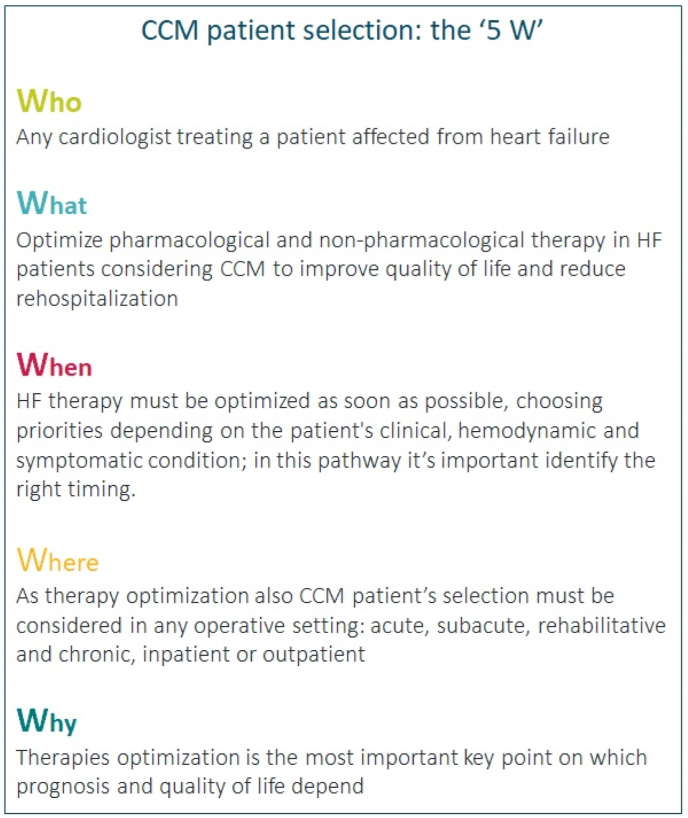
CCM patient selection process summarized in 5 points.

Patient's selection and implantation timing play a crucial role in determining who can get more benefit from CCM therapy; we can summarize in 5 points (called “5 W”) the patient selection process (Fig. 3).

CR could be the best selection pathway for CCM therapy candidates, anticipating the timing of implantation and promoting either the up-titration and the optimization of drugs and CCM optimal management, which already represents the main CR objectives.

## CRediT authorship contribution statement

**Matteo Ruzzolini:** Writing – review & editing, Writing – original draft, Conceptualization. **Francesco Giallauria:** Writing – review & editing, Writing – original draft, Conceptualization. **Francesco Fattirolli:** Supervision. **Elio Venturini:** Supervision. **Francesco Maranta:** Supervision. **Gian Francesco Mureddu:** Supervision. **Pasqualina Calisi:** Supervision. **Raffaele Griffo:** Supervision, daniele masarone, Supervision. **Carlo Vigorito:** Supervision. **Marco Ambrosetti:** Supervision. **Daniele Masarone:** Supervision.
